# Single-cell RNA sequencing highlights the heterogeneity of neutrophils in granulomatosis with polyangiitis

**DOI:** 10.1515/rir-2026-0032

**Published:** 2026-10-22

**Authors:** Shuning Guo, Yangzhong Zhou, Dazhi Pang, Guanqiao Li, Yulin Lyu, Xinyuan Xu, Jing Li, Xinping Tian

**Affiliations:** Department of Rheumatology and Clinical Immunology, Peking Union Medical College Hospital, Chinese Academy of Medical Sciences, Peking Union Medical College, Beijing, China; National Clinical Research Center for Dermatologic and Immunologic Diseases (NCRC-DID), Ministry of Science & Technology, Beijing, China; State Key Laboratory of Complex Severe and Rare Diseases, Peking Union Medical College Hospital, Beijing, China; Key Laboratory of Rheumatology and Clinical Immunology, Ministry of Education, Beijing, China; School of Basic Medical Sciences, Tsinghua University, Beijing, China; Tsinghua Clinical Research Institute, School of Medicine, Tsinghua University, Beijing, China; School of Medicine and Vanke School of Public Health, Tsinghua University, Beijing, China

**Keywords:** granulomatosis with polyangiitis, single-cell sequencing, neutrophil heterogeneity, cell communication

## Abstract

**Background and Objectives:**

Granulomatosis with polyangiitis (GPA), a clinical phenotype of anti-neutrophil cytoplasmic antibody-associated vasculitis, is characterized by necrotizing inflammation of blood vessels. While neutrophil dysregulation is central to its pathogenesis, its cellular heterogeneity and dynamic functional states remain poorly defined. To better understand the transcriptomic profile of neutrophils, we applied single-cell RNA sequencing (scRNA-seq) to peripheral blood samples from GPA patients and healthy controls (HCs).

**Methods:**

Peripheral blood samples were collected from three patients with active GPA and three HCs, followed by sequencing using the BD Rhapsody platform (Becton, Dickinson and Company). In a subsequent validation cohort, flow cytometry and quantitative real-time polymerase chain reaction were used to confirm specific neutrophil subtypes and explore their potential clinical significance.

**Results:**

Seven neutrophil clusters with distinct transcriptomic and functional profiles were identified. CXCL8-high and TXNIP-high subsets, predominant in HCs and GPA patients respectively, showed inflammatory profiles. CXCL8-high subsets also expressed immunoregulatory molecules to restrain inflammation. MMP8-high neutrophils showed enhanced antimicrobial activity, whereas IFIT1-high cells were linked to antiviral responses, suggesting that prior infections may contribute to anti-neutrophil cytoplasmic antibody-associated vasculitis (AAV) pathogenesis. PLA2G7-high neutrophils, identified as terminally differentiated by pseudotime analysis, were associated with disease relapse and renal involvement, indicating potential as AAV biomarkers. Transcriptional profiles of peripheral T and natural killer (NK) cells were also characterized, along with the analysis of systemic immune cell-cell communication networks.

**Conclusion:**

Our study enhances the understanding of the dynamic functional roles of neutrophils in GPA and may facilitate the development of therapeutic strategies aimed at selectively targeting disease-specific neutrophil subsets.

## Introduction

Anti-neutrophil cytoplasmic antibody (ANCA)-associated vasculitis (AAV) manifests as a heterogeneous autoimmune pathology characterized by autoantibodies against neutrophil cytoplasmic components including myeloperoxidase and proteinase 3, leading to small vessel damage and multisystem involvement. AAV encompasses granulomatosis with polyangiitis (GPA), microscopic polyangiitis (MPA) and eosinophilic granulomatosis with polyangiitis.^[1]^ Despite evolving therapies,^[2–4]^ treatment remains largely non-individualized. A thorough understanding of immune characteristics could enable precise prognostication and individualized therapy, potentially transforming vasculitis management. Reliable biomarkers are also urgently needed to predict treatment response and monitor disease progression.

Various pro-inflammatory immune cells contribute to AAV pathogenesis, with neutrophils as key effectors. Persistent ANCA binding activates neutrophils, directing them to micro-vascular sites where they induce damage through reactive oxygen species, degranulation, protease release, and neutrophil extracellular trap (NET) formation *via* NETosis.^[1,5,6]^ Despite the central role of neutrophils, their heterogeneity and functional states remain unclear, and targeted therapies against them are still limited compared to those available for B cells, T cells, or cytokines.^[7]^

Previous studies using flow cytometry and bulk transcriptomics^[8,9]^ were limited by limited marker panels and low sensitivity to subtle, cell-specific transcriptional changes. Single-cell RNA sequencing (scRNA-seq) has enabled higher-resolution analysis of immune alterations.^[10, 11, 12]^ Recent scRNA-seq studies in patients with new-onset MPA have identified an expanded population of interferon-II-responsive neutrophils, driven by interferon-γ (IFN-γ) and TNF signaling, as a potential marker of relapse risk in small vessel vasculitis.^[13]^ However, the molecular features of neutrophils in GPA at single-cell resolution and their role in driving inflammation remain unclear.

To address this challenge, we applied scRNA-seq to a total of 38, 958 peripheral blood cells from three GPA patients with active disease and three healthy controls (HCs). Seven neutrophil subpopulations with distinct transcriptional profiles and functional characteristics were identified. Notably, the *PLA2G7*-high neutrophils (Neu_PLA2G7) subpopulation emerged as a potential biomarker for AAV, given its association with clinical features. Our study sheds new light on the pathogenesis of GPA and suggests novel targets for therapeutic intervention.

## Methods

### Human Subjects

Patients with GPA or MPA and HCs were enrolled from Peking Union Medical College Hospital. Patients diagnosed with GPA or MPA fulfilled both the 2012 Chapel Hill Consensus Conference definitions and the 1990 American College of Rheumatology Classification Criteria.^[14,15]^ The disease activity was evaluated using the Birmingham Vasculitis Activity Score (BVAS) 3.0 version. Remission was defined as the absence of active disease manifestations (BVAS = 0). Exacerbation was characterized by the emergence of new or recurrent signs and symptoms (BVAS > 6), which necessitated intensified immunosuppressive therapy in accordance with the recommendations from the European Alliance of Associations for Rheumatology.^[16]^ Patients with a prior diagnosis of cancer, end-stage renal disease requiring dialysis, or infection were excluded from the study. All samples were collected in compliance with the Declaration of Helsinki and approved by the Ethics Committee of Peking Union Medical College Hospital (No. S-478). All participants provided written informed consent. Supplementary Table 1 contains detailed participant information.

### Library Preparation and Sequencing

Peripheral blood from three GPA patients and three HCs was analyzed by scRNA-seq using the BD Rhapsody platform (Becton, Dickinson and Company). Whole transcriptome libraries were prepared following the BD Rhapsody workflow and sequenced on the Illumina HiSeq Xten platform with 150 bp paired-end reads.

### ScRNA-seq Data Processing

The scRNA-seq data were processed using Seurat (v4.3.0) in R (v4.4.2). Cells were filtered by the following criteria: gene count < 200 or > 4000, total transcripts < 1000 or > 97th percentile, mitochondrial content > 15%, and hemoglobin genes > 5%. After normalization, cell cycle scores (S and G2/M phases) were calculated using canonical markers and regressed out during data scaling to reduce confounding effects. Dimensionality reduction and batch correction were performed *via* Harmony (RunHarmony), and uniform manifold approximation and projection (UMAP) was used to visualize major cell subsets. Clustering was performed at a resolution of 0.03, and the top 30 differentially expressed genes (DEGs) per cluster were identified based on known marker genes. Two independent researchers annotated and validated cell identities.

Subclustering was conducted for neutrophils, T cells, natural killer (NK) cells, and myeloid cells, followed by re-integration and clustering using the same pipeline. RunHarmony corrected batch effects for neutrophils, while canonical correlation analysis (CCA) was used for T/NK and myeloid cells. Neutrophils, T/NK cells and myeloid cells were clustered using highly variable genes, with resolutions set at 0.2, 0.5 and 0.1, respectively. Cell proportions were compared using unpaired two-tailed Student’s t-tests. DEGs were identified *via* FindAllMarkers (adjusted *P* < 0.05, expression > 10%) and ranked by mean log fold-change. Top 50 genes per cluster were used for visualization. Functional enrichment of DEGs was performed *via* Metascape,^[17]^ which integrates current biological databases including Gene Ontology (GO), Kyoto Encyclopedia of Genes and Genomes (KEGG), Reactome Pathway Database, Wikipathways, and CORUM databases. Neutrophil trajectories were reconstructed using Monocle 2 for pseudotime analysis.^[18]^ CellChat was applied to map intercellular communication networks among immune subsets based on the standard workflow.^[19]^

### Flow Cytometry

Peripheral blood was collected from three GPA patients and three HCs. Neutrophils were isolated utilizing the Human Peripheral Blood Neutrophil Isolation Kit (Solarbio, China), then stimulated with eBioscience™ Cell Stimulation Cocktail (plus protein transport inhibitors) (500X) (Thermo Fisher Scitific, USA) at 37°C in 5% CO_2_. For intracellular staining, cells were fixed and permeabilized (Cytofix/Cytoperm and Perm/Wash Buffer, BD Biosciences, USA), then incubated with PLA2G7 polyclonal antibody at 37°C in 5% CO_₂_ for 1.5 h, followed by FITC-conjugated goat anti-rabbit IgG (Proteintech, China) for 1 h. Stained neutrophils were analyzed on a BD FACSAria™ II flow cytometer, and data processed using FlowJo v10.8.

### Reverse Transcription Quantitative Polymerase Chain Reaction (RT-qPCR)

Peripheral venous blood was obtained from eighteen AAV patients (including eleven GPA and seven MPA) and eighteen HCs. Neutrophils were isolated as described above. The RNA extraction, reverse transcription and quantative PCR were performed following established protocols.^[20]^ The primer sequences of *PLA2G7* were: 5’-TGGCTCTACCTTAGAACCCTGA-3’ for the forward primer and 5’-TTTTGCTCTTTGCCGTACCT-3’ for the reverse primer. Relative gene expression was calculated using the 2−ΔΔCq method.

### Statistical Analysis

GraphPad Prism (version 10.2.1) was used for all statistical analyses. Visualizations were generated utilizing R software (version 4.4.2) and GraphPad Prism (version 10.2.1). Between-group comparisons were performed using unpaired two-sided *t*-tests, and associations between PLA2G7 expression and clinical indicators were assessed using simple linear regression.

## Results

### An Overview of Peripheral Blood Cells in Patients with GPA

Peripheral blood samples were obtained from three patients with GPA and three HCs. All GPA patients were in active disease, with two patients being newly diagnosed. Clinical details are provided in Supplementary Table 1. We performed scRNA-seq on isolated peripheral blood cells using the BD Rhapsody platform (Figure 1A), yielding 38, 958 high-quality cells after quality filtering (Supplementary Figure S1A), with 51.6% from GPA patients and 48.4% from HCs (Supplementary Figure S1B-D). Following batch effect correction, dimensionality reduction, and graph-based clustering, five major immune cell lineages were identified (Figure 1B and C), including neutrophils (*FCGR3B*+), T/NK cells (*CD3D*+ or *KLRD1*+), B cells (*CD79A*+), myeloid cells (*CD14*^+^) and basophils (*FCER1A*+), as validated by canonical lineage markers and cluster-specific expression patterns (Figure 1D and E). GPA patients exhibited a marked increase in neutrophil proportion compared to HCs (Supplementary Figure S1E).

**Figure 1:**
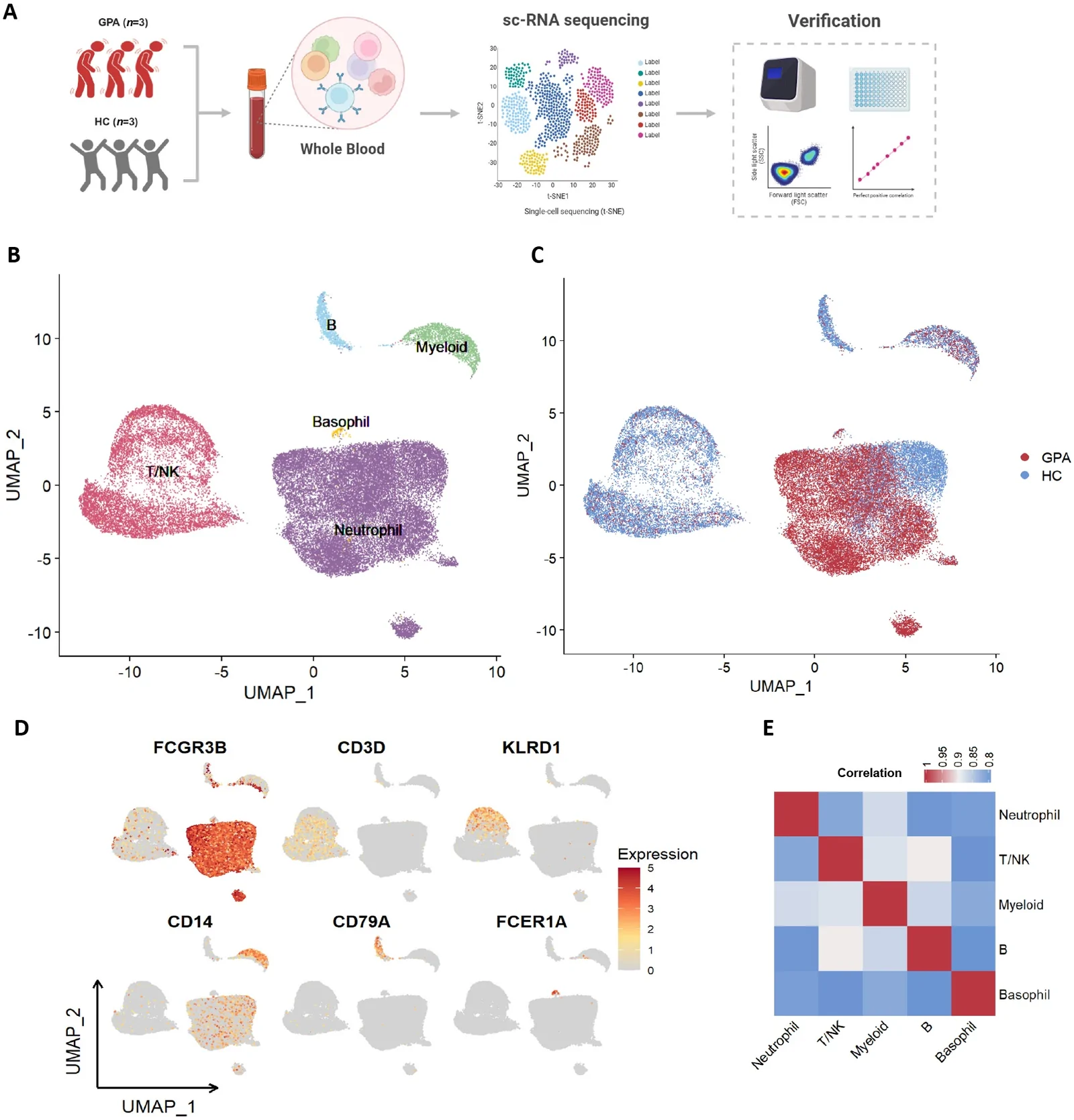
High-resolution mapping of peripheral blood cells in GPA patients and healthy controls. (A) Overview of the integrated experimental workflow. (B) UMAP visualization depicting five distinct cell clusters (colored) identified in the peripheral blood from three GPA patients and three HCs. (C) UMAP plot colored by group. (D) UMAP plot displaying the expression of canonical markers for the major cell lineages. (E) Heatmap showing the Spearman correlations (colors) across all cell types based on log-normalized average expression. UMAP, uniform manifold approximation and projection; GPA, granulomatosis with polyangiitis; HCs, healthy controls.

### The Identification of Distinct Transcriptomic Characteristics of Neutrophils

Although neutrophil activation is pivotal in AAV pathogenesis, the heterogeneity of neutrophils remains unexplored. Therefore, we focused on the distinct characteristics of neutrophils and investigated their contributions to pathogenesis in GPA. Among 38,958 single cells, 28,907 neutrophils (74.1%) were identified, with a median of 790 genes detected per cell (Supplementary Figure S2A). Seven distinct clusters were identified (Figure 2A), which were not influenced by sample variation (Supplementary Figure S2B), and validated by known gene signatures.^[21, 22, 23]^ The top 30 upregulated genes in neutrophil subtypes from GPA patients compared to HCs were listed in Supplementary Table 2. We observed significant differences in the proportions of several neutrophil subtypes between GPA patients and HCs (Figure 2E).

**Figure 2:**
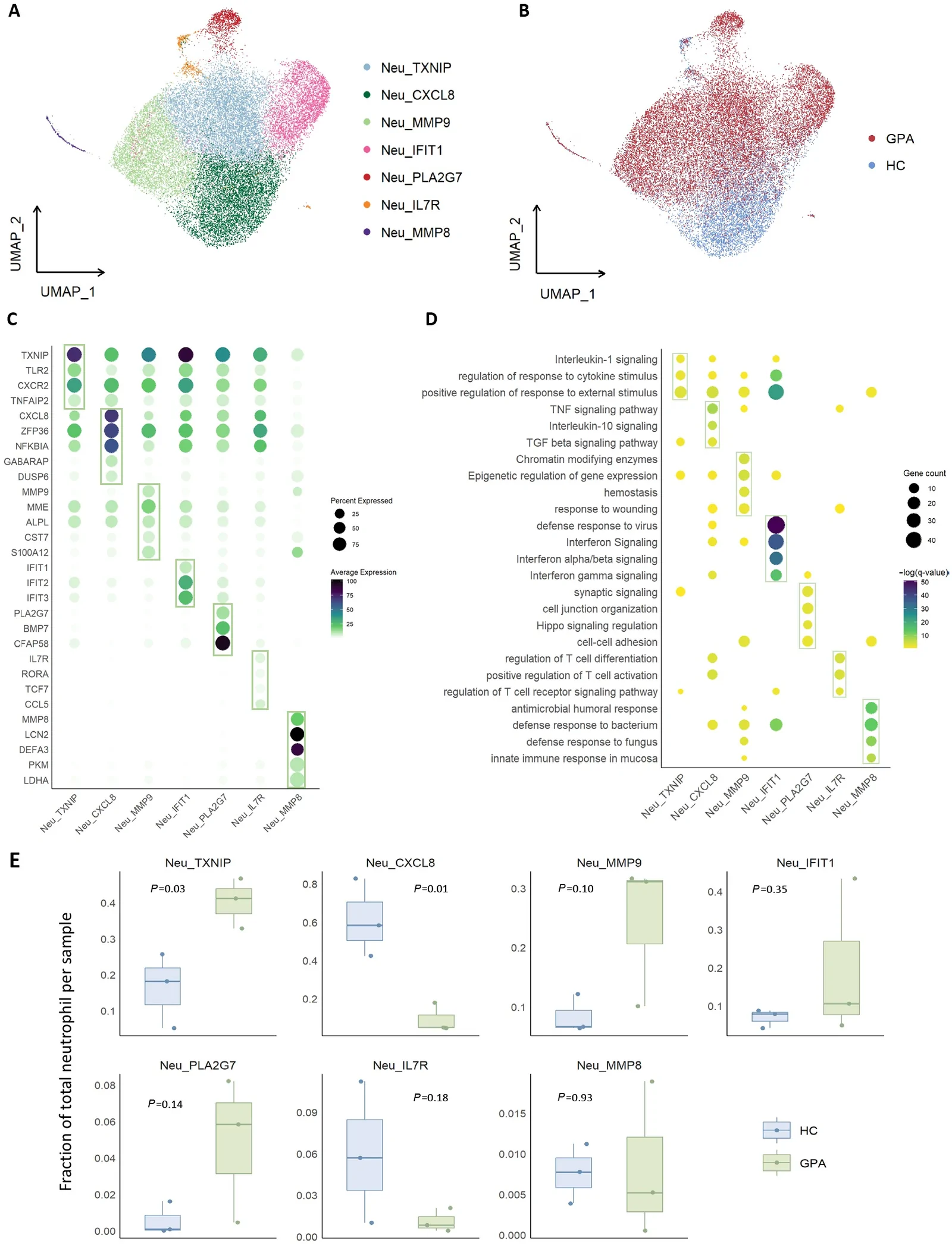
Heterogeneity of neutrophil subtypes in GPA. (A) UMAP plot of circulating neutrophils of GPA patients (n = 3) and HCs (n = 3), colored according to the identified subpopulations. (B) UMAP plot colored by group. (C) Dot plot illustrating the expression levels of highly expressed genes across seven cell subpopulations. The size of each circle represents the proportion of cells within the subpopulation that express the gene, and the color denotes the average expression level. (D) Representative pathways enriched by the highly expressed genes in neutrophil subpopulations, identified from five databases: GO, KEGG, Reactome Pathway Database, Wikipathways, and CORUM. The size of each circle reflects the number of genes within the corresponding pathways, and the color represents the-log10 transformed Benjamini-Hochberg corrected P-value. (E) Neutrophil subpopulation proportions in GPA patients and HCs (center lines, medians; dots, individual participants; unpaired two-sided t-tests). UMAP, uniform manifold approximation and projection; GPA, granulomatosis with polyangiitis; HCs, healthy controls; GO, Gene Ontology; KEGG, Kyoto Encyclopedia of Genes and Genomes.

The diversity of neutrophils corresponds to distinct functional specializations. We analyzed the high-expression genes and enriched pathways of all seven subpopulations (Figure 2C and D). Specifically, *TXNIP*-high neutrophils (Neu_TXNIP) were associated with inflammation-related genes (*TLR2*, *CXCR2* and *TNFAIP2*) and showed enrichment in inflammatory responses including interleukin-1 signaling. Similarly, *CXCL8*-high neutrophils (Neu_CXCL8) were enriched in inflammatory responses, including the TNF signaling pathway, interleukin-10 signaling and TGF beta signaling. This subtype also upregulated immune suppression molecules (such as *ZFP36*, *NFKBIA*, *GABARAP* and *DUSP6*) to regulate excessive immune reactions. *IFIT1*-high neutrophils (Neu_IFIT1) displayed pronounced interferon-stimulated profiles, with strong upregulation of interferon-responsive genes (IFIT1, IFIT2, IFIT3) and enrichment in interferon signaling. Moreover, *MMP8*-high neutrophils (Neu_MMP8) were enriched in antibacterial genes (*LCN_2_*, *DEFA3*, *PKM* and *LDHA*) and antimicrobial pathways. *MMP9*-high neutrophils (Neu_MMP9) up-regulated tissue repair-related genes (such as *MME*, *ALPL*, and *CST7*), and were associated with epigenetic regulatory pathways. *IL7R*-high neutrophils (Neu_IL7R) were closely linked to the regulation of T cell activation. *PLA2G7*-high neutrophils (Neu_PLA2G7) were enriched in synaptic function and adhesion-related pathways. These findings indicate that each neutrophil subpopulation exhibits unique features and contributes to the pathogenesis of GPA.

We compared neutrophil subtype composition between GPA patients and HCs and found a significant increase in Neu_TXNIP (*P* = 0.03) and a decrease in Neu_CXCL8 (*P* = 0.01) in GPA (Figure 2E), suggesting these subtypes may predominate in GPA and HCs, respectively. Neu_IFIT1, Neu_ PLA2G7, and Neu_MMP9 also showed upward trends in GPA.

However, these trends did not reach statistical significance, likely attributable to the limited sample size of the present cohort. Further validation in a larger cohort will be essential to confirm the reliability of these observations. These findings indicate substantial shifts in neutrophil composition and function in GPA, reflecting immune microenvironment remodeling.

### Identification of Functional Characteristics of Neu_ PLA2G7

Pseudotime analysis was performed to investigate the differentiation trajectories of neutrophil subtypes in GPA patients (Figure 3A). The trajectory revealed a clear progression of neutrophils, with cells transitioning from early to late stages along the pseudotime axis (Supplementary Figure S3A and B). Compared to HCs, neutrophils from GPA patients were enriched at later pseudotime stages (Figure 3B), reflecting a more activated and dynamic immune profile. At the first branch point, two distinct trajectories emerged (Figure 3A, Supplementary Figure S3B), suggesting divergent developmental or activation pathways. Neu_TXNIP, Neu_CXCL8, Neu_IFIT1 and Neu_MMP9 were broadly distributed along the pseudotime axis (Figure 3C), highlighting their critical roles in neutrophil function, immune coordination, and inflammation regulation throughout all stages of development or activation. In contrast, Neu_PLA2G7 were primarily found at the end of one trajectory (fate 2; Figure 3C), indicating a mature neutrophil state that may represent the final stage of activation in GPA.

**Figure 3:**
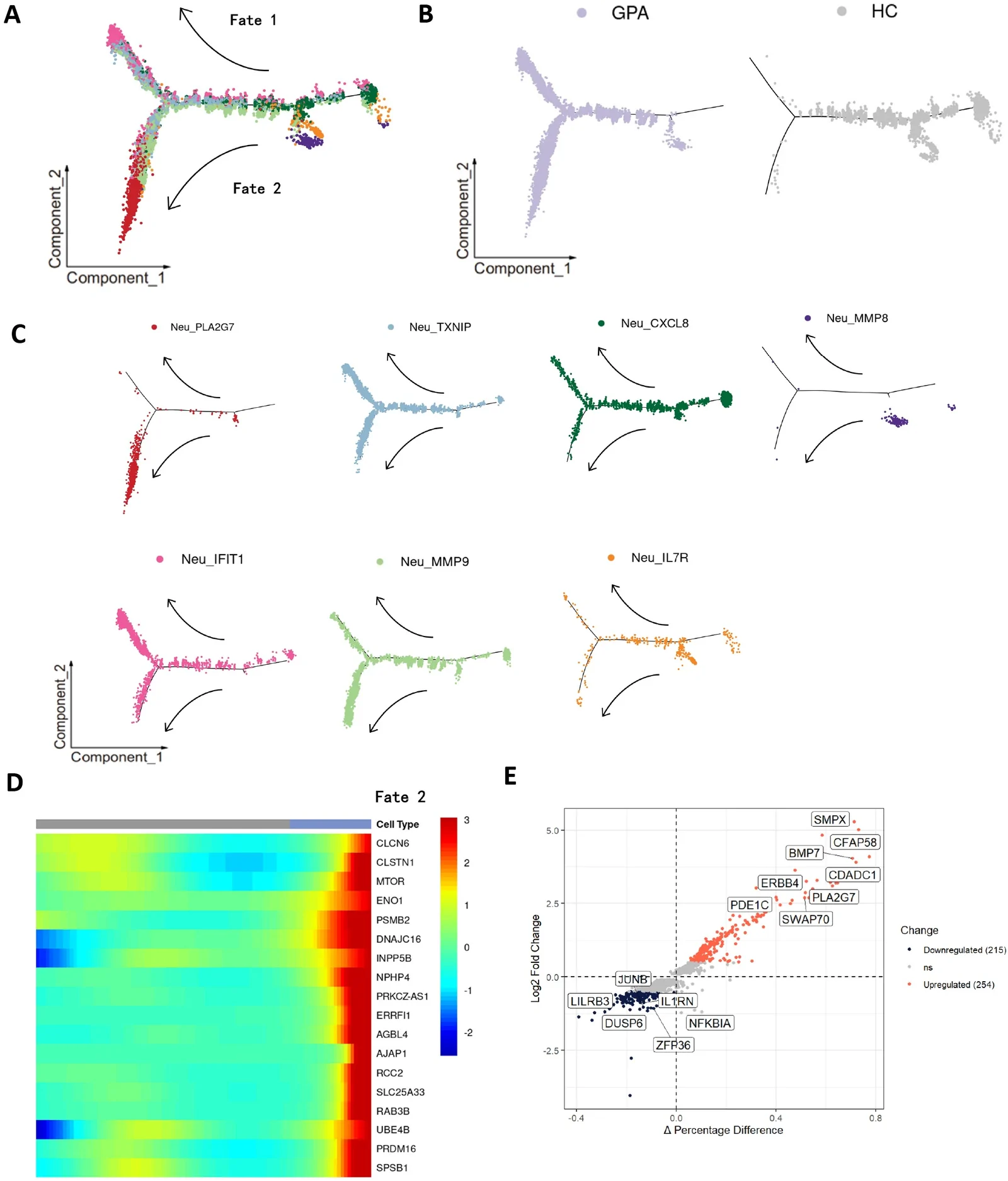
Trajectory inference of neutrophil differentiation. (A) Pseudo-time trajectory of neutrophil clusters, with each dot representing an individual cell. (B) Pseudo-time trajectory of neutrophils, colored by group. (C) Pseudo-time trajectory of neutrophils, colored by neutrophil subtype. (D) Heatmap depicting gene expression (normalized log count, shown by color) of correlated genes along fate 2. (E) Volcano plot showing differentially expressed genes of Neu_PLA2G7. The y-axis represents the log2 fold change in gene expression, and the x-axis represents the percentage difference across clusters. GPA, granulomatosis with polyangiitis; HC, healthy control; Neu_PLA2G7, PLA2G7-high neutrophils.

We next examined gene expression along the two differentiation trajectories. In fate 2, genes involved in adhesion and migration (including *CLSTN1*, *INPP5B*, *NPHP4*, *AGBL4*, *AJAP1*, *RCC2*) showed increased expression (Figure 3D), suggesting their involvement in the late-stage activation of Neu_PLA2G7. Differential expression analysis identified 254 upregulated and 215 downregulated genes in this subpopulation (Figure 3E). Upregulated genes such as *CDADC1*, *CFAP58* and *SWAP70* indicate potential roles in migration and intercellular communication, while *BMP7*, *ERBB4*, and *PDE1C* are associated with differentiation and cellular growth. Elevated *PLA2G7* expression highlights its role in lipid metabolism in AAV, as it hydrolyzes oxidized LDL into pro-inflammatory lipids that promote immune activation and oxidative stress.^[24]^ Conversely, several genes involved in immune regulation and inflammatory responses (*LILRB3*, *DUSP6*, *ZFP36*, *NFKBIA*, *IL1RN* and *JUNB*) were downregulated, indicating that Neu_PLA2G7 may contribute to GPA pathogenesis indirectly by modulating other immune cells, rather than through its own pro-inflammatory gene expression.

### Neu_PLA2G7 As A PotentiAl BiomArker in AAV

Given the distinct functional state of the Neu_PLA2G7 subset, we further explored its potential role in patients with AAV. Flow cytometry confirmed the presence of this subset in GPA samples (Figure 4A). Due to the difficulty of staining intracellular proteins in neutrophils, we next examined the RNA expression levels of *PLA2G7* between the two groups. Consistently, peripheral neutrophils from AAV patients exhibited significantly higher *PLA2G7* RNA levels (GPA versus HC, *P* = 0.0007; MPA versus HC, *P* = 0.0064; Figure 4B). ROC analysis supported its diagnostic potential, with an AUC of 0.747 (*P* = 0.011; Supplementary Figure S4A). Notably, *PLA2G7* expression was higher in GPA patients than in MPA patients (*P* = 0.0314; Figure 4B).

**Figure 4:**
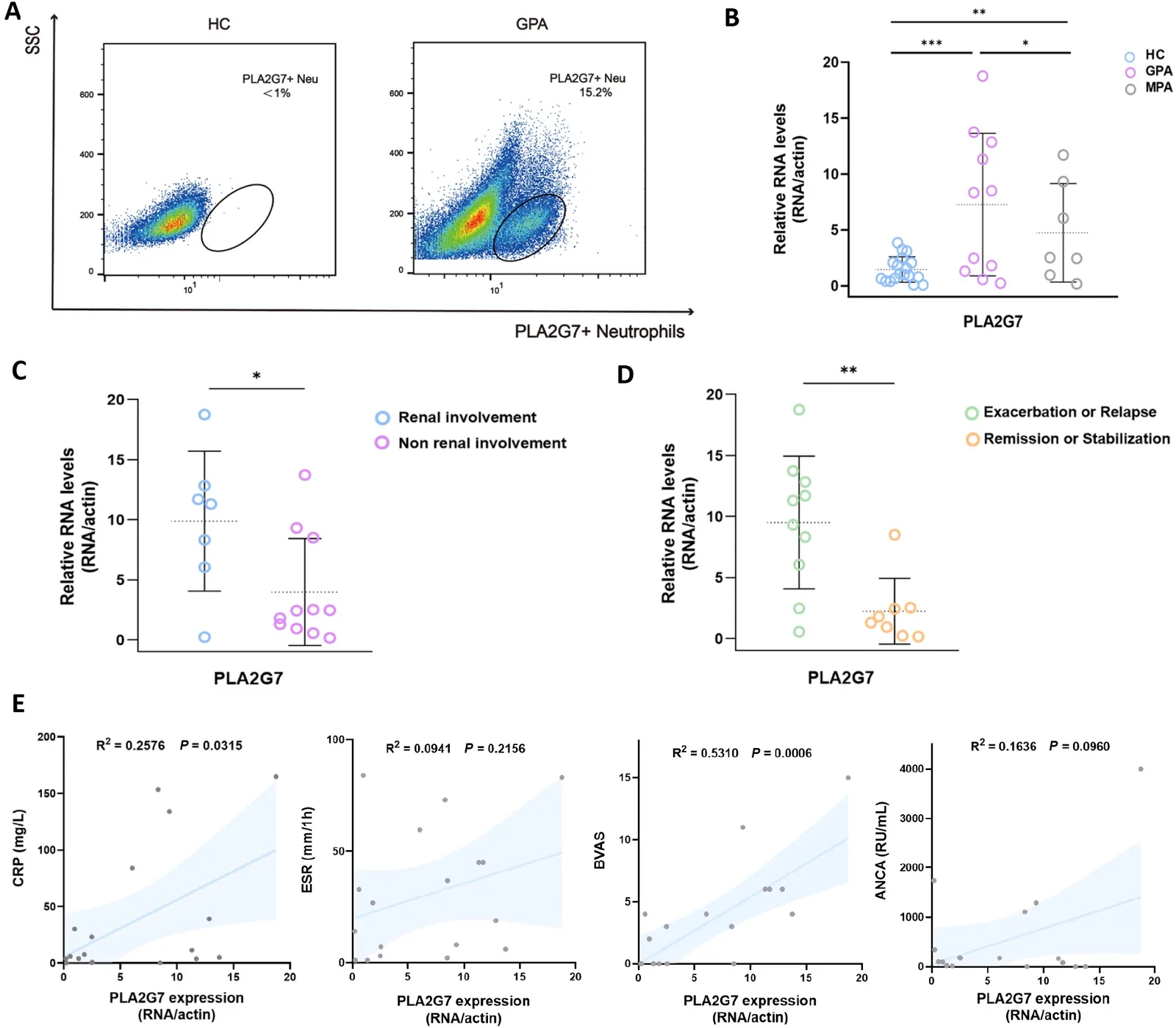
Identification of the Neu_PLA2G7 subpopulation as potential biomarkers. (A) Representative flow cytometry plot of Neu_PLA2G7 subpopulations in GPA patients and HCs (n = 3 in HC group, n = 3 in GPA group). (B) Relative RNA levels of PLA2G7 in neutrophils, as measured by RT-qPCR, from AAV patients and HCs (n = 18 in HC group, n = 11 in GPA group, n = 7 in MPA group). (C) Relative RNA levels of PLA2G7 in neutrophils from AAV patients with and without renal involvement. (D) Relative RNA levels of PLA2G7 in neutrophils from patients in exacerbation/relapse versus remission/stabilization. (E) Correlation between PLA2G7 expression in neutrophils and clinical indicators in AAV patients. Data were presented as means ± standard deviation, and p-values were calculated by unpaired two-sided t-tests and simple linear regression analysis. *P ≤ 0.05; **P ≤ 0.01; ***P ≤ 0.001. Neu_PLA2G7, PLA2G7-high neutrophils; GPA, granulomatosis with polyangiitis; HCs, healthy controls.

In our analysis, we also observed elevated *PLA2G7* expression in neutrophils from patients with renal involvement. Accordingly, stratifying patients revealed significantly higher *PLA2G7* RNA expression in those with kidney involvement (Figure 4C and Supplementary Figure S4B). To explore the relationship between *PLA2G7* expression and disease activity in AAV, we stratified patients by clinical status. *PLA2G7* expression was significantly higher in patients experiencing disease exacerbation or relapse compared to those in remission or stabilization (Figure 4D, *P* = 0.0033). ROC analysis yielded an AUC of 0.875 (*P* = 0.008; Supplementary Figure S4C), suggesting strong predictive value for disease flares. Correlation analysis showed *PLA2G7* expression positively associated with C-reactive protein (CRP; R^2^ = 0.2576, *P* = 0.0315; Figure 4E) and strongly correlated with BVAS scores (R^2^ = 0.531, *P* = 0.0006; Figure 4E), underscoring its relevance to disease severity. These findings support *PLA2G7* expression levels in neutrophils as a potential biomarker for monitoring vasculitis progression, particularly in renal involvement.

### Transcriptional Features of T/NK-cell and Other Myeloid Subpopulations in GPA

We next focused on T/NK and other myeloid subpopulations. T cells were divided into eight subsets based on canonical marker gene expression:^[25, 26, 27]^ naïve CD4^+^ T cells (CD4T_Tn_CCR7, *IL4R*, *FOXP1*, *CCR7*, *TCF7*, *LEF1* and *SELL*), memory CD4^+^ T cells (CD4T_Tm_IL7R, *IL7R*, *S1PR1*, *PTGER4* and *GPR183*), regulatory CD4^+^ T cells (CD4T_Treg_FOXP3, *FOXP3*, *IL2RA* and *RTKN_2_*), naïve CD8^+^ T cells (CD8T_Tn_CCR7, *FOXP1*, *CCR7*, *TCF7*, *LEF1* and *SELL*), effector memory CD8^+^ T cells (CD8T_Tem_GZMK, *GZMK*, *CXCR4* and *CXCR3*), mucosal-associated invariant CD8^+^ T cells (CD8T_MAIT_SLC4A10, *SLC4A10*, *KLRB1* and *RORC*), natural killer CD8^+^ T cells (CD8T_NK_like_FGFBP2, *FGFBP2*, *GZMB* and *NKG7*) and γδ T cells (γδ T_TRDC, *TRDC*) (Figure 5A and B, Supplementary Figure S5A). Due to the limited sample size, no statistically significant differences were detected between the GPA and HC groups in the scRNA-seq data (Figure 5C). Functional analysis revealed that CD4T_Tm_IL7R, CD8T_Tem_GZMK, and CD8T_MAIT_SLC4A10 subpopulations were enriched in the TNF signaling pathway, with the latter two also showing enrichment in the IL-17 signaling pathway (Figure 5D). CD4T_Tn_CCR7 showed enrichment in the PI3K Akt signaling and the JAK-STAT signaling pathway (Figure 5D). These pathways represent key therapeutic targets, providing mechanistic support for the use of TNF inhibitors, IL-17 inhibitors, JAK inhibitors, and mTOR pathway modulators such as rapamycin.

**Figure 5:**
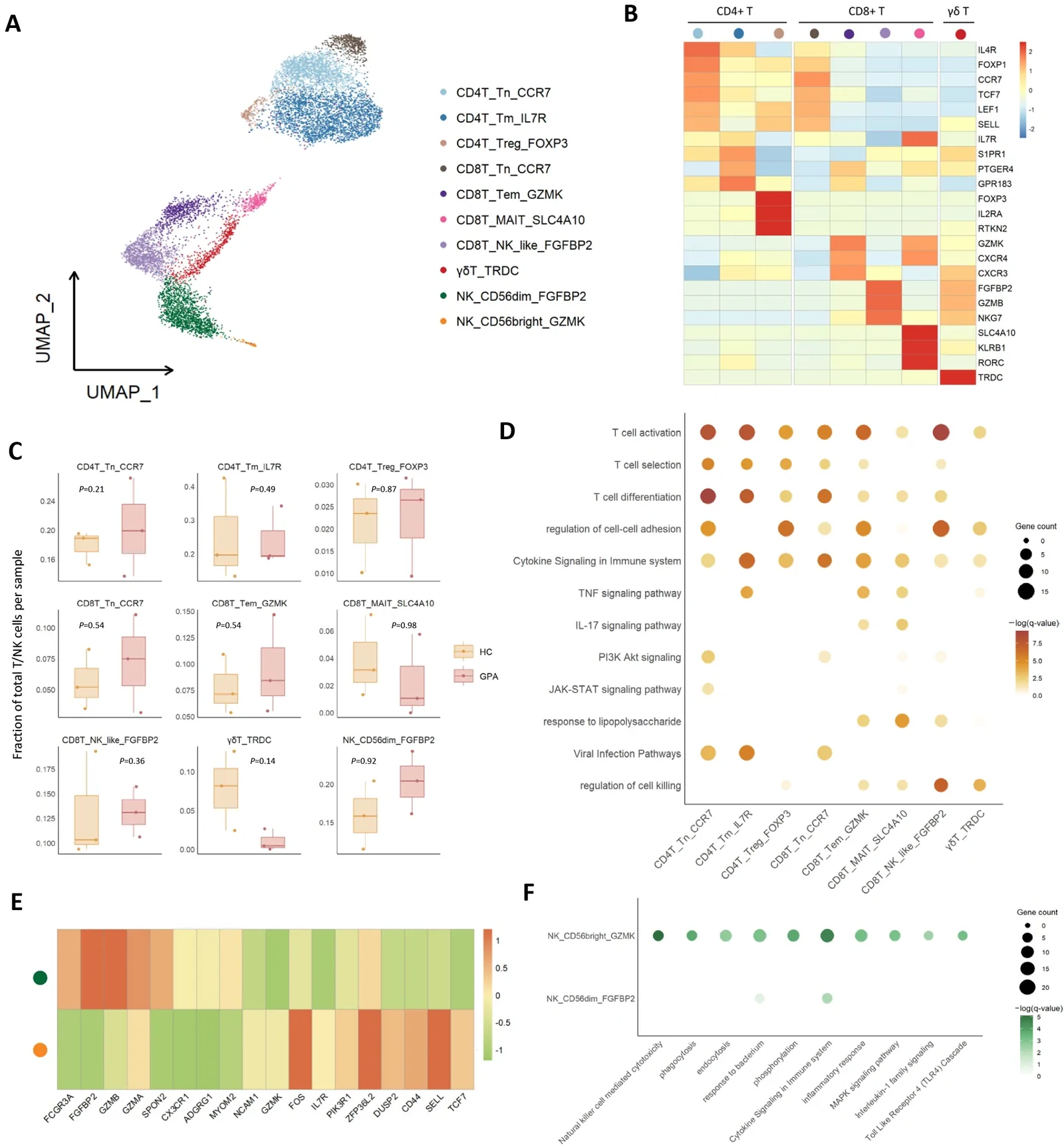
Heterogeneity of T/NK subtypes in GPA. (A) UMAP plot depicting the distribution of circulating T/NK cells in GPA patients (n = 3) and HCs, (n = 3), with color coding based on the identified subpopulations. (B) Heatmap showing the normalized average expression of T cell function-associated genes across each cell subpopulation. (C) T/NK subpopulation proportions in GPA patients and HCs (center lines, medians; dots, individual participants; unpaired two-sided t-tests). (D and F) Representative pathways enriched by the highly expressed genes in T (D) and NK subpopulations (F), identified from five databases: GO, KEGG, Reactome Pathway Database, Wikipathways, and CORUM. The size of each circle reflects the number of genes within the corresponding pathways, and the color represents the-log10 transformed Benjamini-Hochberg corrected p-value. (E) Heatmap showing the normalized average expression of NK cell function-associated genes in each cell subpopulation. UMAP, uniform manifold approximation and projection; T/NK, T cells and natural killer cells; GPA, granulomatosis with polyangiitis; HCs, healthy controls; GO, Gene Ontology; KEGG, Kyoto Encyclopedia of Genes and Genomes.

NK cells were subdivided into two subtypes based on canonical markers:^[28]^ CD56 dim NK cells (NK_CD56dim_FGFBP2, *FCGR3A*) and CD56 bright NK cells (NK_CD56bright_GZMK, *NCAM1*). NK_CD56dim_FGFBP2 displayed elevated levels of cytotoxic molecules (*GZMA* and *GZMB*) and maturation markers (*ADGRG1* and *SPON_2_*). In contrast, NK_CD56bright_GZMK showed strong GZMK expression, along with elevated levels of migration- and adhesion-related genes (*CD44*, *SELL* and *IL7R*) and transcription factors (*FOS* and *TCF7*) (Figure 5E). Functional enrichment analysis revealed that NK_CD56bright_GZMK exhibited greater activity compared to NK_CD56dim_FGFBP2, including cytotoxicity, phagocytosis, endocytosis and proinflammatory responses (such as cytokine signaling in immune system, inflammatory response, and interleukin-1 family signaling) (Figure 5F). Moreover, the myeloid cells were divided into CD14^+^ monocytes (CD14_Mono), CD16^+^ monocytes (CD16_Mono) and dendritic cells (DC) (Supplementary Figure S5B), with their marker gene profiles shown in Supplementary Figure S5C.

### Global Analysis of Immune Cell Communications in GPA

To explore peripheral immune cell interactions, CellChat was employed to construct a cell-cell communication network based on established ligand-receptor pairs and cofactors.^[19]^ CD14_Mono and CD16_Mono showed the highest interaction frequencies, indicating strong signaling activity (Supplementary Figure S6A). CD8^+^T cells exhibited stronger communication than CD4^+^T cells (Supplementary Figure S6A). Specifically, CXCL signaling was the predominant pathway mediating neutrophil interactions (Figure 6A). Among neutrophil subsets, Neu_TXNIP, Neu_MMP9 and Neu_IFIT1 most frequently signaled to CD8^+^ T cells, mainly *via* MHC-I (Figure 6A, Supplementary Figure S6B). Additionally, galectin signaling pathways were involved in these neutrophil subpopulations (Figure 6A). CD14_Mono primarily communicates with neutrophils *via* the SEMA4 pathway, as well as through ANXA1-FPR1 and LGALS9-CD45 pathways (Figure 6A, Supplementary Figure S6C).

**Figure 6:**
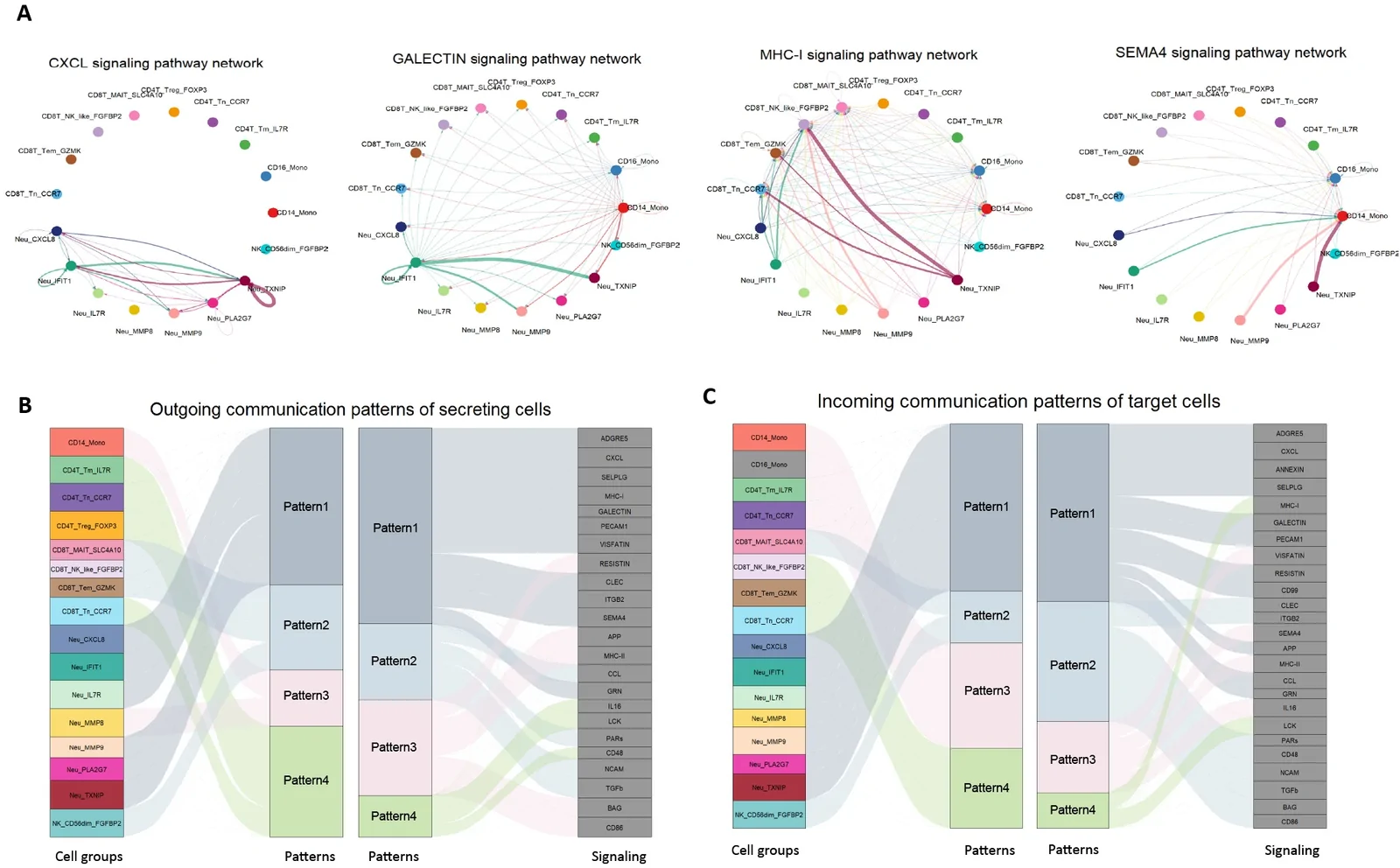
CellChat analysis of immune cell interactions. (A) Circle plot representing the inferred intercellular communication network for CXCL, GALECTIN, MHC-I, and SEMA4 signaling across various cell subsets. The size of each circle corresponds to the number of cells in each cluster, while the line thickness indicates the strength of the signaling. (B) Inferred outgoing communication profiles from secretory cells, showing the correlation between latent patterns, cell populations, and signaling pathways. The flow thickness reflects the contribution of cell subsets or signaling pathway to the corresponding latent pattern. (C) Inferred incoming communication profiles for target subsets.

We further analyzed the flow and dynamics of signaling pathways and integrated cell-cell communication networks using a river plot (Figure 6B and C). Neutrophil signaling was largely driven by pattern #1, involving ADGRE5, CXCL, and PECAM1 pathways (Figure 6B). Neu_MMP8 and CD14_Mono shared RESISTIN, APP, and MHC-II signaling (Figure 6B). Pattern #4, characterized by IL16, LCK, and CD48, was prominent in CD4^+^ T cells and CD8T_Tn_CCR7, while most CD8^+^ T subsets and NK_CD56dim_FGFBP2 showed distinct signals like CCL, PARs, NCAM, and TGFb. The patterns of incoming and outgoing signals differed. Neutrophils were primarily involved in signaling through ADGRE5, CXCL, and ANNEXIN (Figure 6C). CD8^+^ T subpopulations received MHC-I and LCK, while monocytes and CD4^+^ T cells received VISFATIN, SEMA4, and MHC-II (Figure 6C). These findings highlight the heterogeneity of immune communication in GPA, suggesting dysregulated signaling and potential therapeutic targets.

## Discussion

AAV, comprising mainly GPA and MPA, is a severe autoimmune vasculitis. Using scRNA-seq, we revealed the transcriptomic and functional heterogeneity of peripheral neutrophils in GPA, identifying seven distinct clusters. Notably, Neu_PLA2G7 was closely linked to disease relapse and renal involvement, highlighting its potential as a biomarker. To our knowledge, this is the first scRNA-seq study to characterize neutrophil heterogeneity in GPA.

Recent scRNA-seq and surface antigen profiling of neutrophils from patients with new-onset MPA delineated seven distinct neutrophil subtypes.^[13]^ Among them, the Neu_T2ISG subset, characterized by type II interferon signature genes, was found to be expanded and implicated in promoting myeloperoxidase induction, Fcγ receptor expression, and ANCA-induced neutrophil extracellular trap formation.^[13]^ AAV is widely recognized to be triggered by prior infections, and in MPA patients, those with a longer interval from the onset of vasculitis symptoms exhibit a higher proportion of the Neu_T2ISG subset.^[13]^ In our dataset, we identified a phenotypically similar subpopulation, Neu_IFIT1, marked by strong activation of both type I and type II interferon pathways. A previous study also highlighted that GPA is characterized by a Th1-related cytokine profile, particularly enriched in IFN-γ. ^[29]^ These observations imply that prolonged exposure to pathogenic agents may drive sustained myelopoiesis, thereby facilitating the expansion of long-lived subsets, including Neu_T2ISG in MPA patients and Neu_IFIT1 in our dataset. Notably, IFN-γ, a cytokine that typically exerts an antagonistic effect on myelopoiesis,^[30]^ might promote the emergence of pathogenic neutrophil subsets in AAV patients. Besides, this subset was observed not only under inflammatory conditions but also frequently in the tumor microenvironment,^[21,22]^ suggesting the presence of a conserved interferon-driven neutrophil activation program across distinct disease contexts.

In addition to Neu_IFIT1, Neu_CXCL8 and Neu_TXNIP, which are predominant in HCs and GPA patients respectively, exhibit distinct inflammatory profiles. Neu_CXCL8 also express immunoregulatory molecules to restrain excessive immunity and maintain homeostasis in HCs, whereas Neu_TXNIP may drive GPA-associated immune activation. Furthermore, Neu_ MMP8 and Neu_MMP9 subsets in our dataset exhibit similar gene expression profiles to Myelocyte and Neu_Immature subtypes in MPA patients.^[13]^ These two subsets in our dataset are enriched at early pseudotime stages, reflecting their non-activated, immature state. Neu_MMP8 is implicated in antibacterial responses. Neu_MMP9 is found to upregulate tissue repair-related genes, suggesting involvement in post-injury repair processes. Neu_IL7R is closely linked to the regulation of T cell activation, indicating potential crosstalk with adaptive immunity. Neu_PLA2G7 is enriched in synaptic function and adhesion-related pathways, which may facilitate enhanced intercellular crosstalk among immune cells. These findings indicate that each neutrophil subpopulation exhibits unique features and contributes to the pathogenesis of GPA.

Our study highlighted the clinical relevance of the Neu_ PLA2G7 subtype as a potential biomarker in AAV. *PLA2G7* RNA levels in neutrophils were elevated in AAV patients and were associated with disease exacerbation, relapse, and renal involvement. *PLA2G7*, a member of the phospholipase A2 family, has been implicated in kidney pathology through receptor-mediated pathways, particularly in idiopathic membranous nephropathy.^[31,32]^ Additionally, *BMP7*, which is also elevated in Neu_PLA2G7, is a key member of the TGF-β family essential for cellular differentiation, and its deletion leads to early-onset uremia in mice.^[33]^ These findings support Neu_PLA2G7 subset as a potential indicator of renal involvement in AAV. Moreover, *PLA2G7* has been identified as a key regulator of inflammatory macrophage polarization in experimental autoimmune encephalomyelitis models and multiple sclerosis patient-derived monocytes.^[34]^ Murine *PLA2G7* deletion mitigated age-related thymic lipoatrophy, attenuated NLRP3 inflammasome activation, and improved metabolic homeostasis.^[35,36]^ In neutrophils, PLA2G7 influences FOXO3-related functions in inflammatory bowel disease and inflammation-linked metabolism.^[37]^ Strong *PLA2G7* expression has also been reported in LPS-exposed rat granulocytes.^[38]^ However, in our study, Neu_PLA2G7 did not display a strong inflammatory signature, but rather showed downregulated genes involved in immune regulation and inflammation. The upregulated genes were associated with enhanced migration and communication abilities, suggesting that Neu_PLA2G7 may contribute to inflammation by activating other neutrophil subsets or immune cells. Further investigations into the functions and mechanisms of the Neu_PLA2G7 subset are essential for understanding the role of neutrophils in AAV, especially in renal involvement.

We aimed to map the peripheral immune landscape and intercellular communication, with a particular focus on the understudied neutrophil population. Recent scRNA-seq studies have explored immune cell profiles in AAV. Masayuki *et al*. identified two MPA patient subgroups based on monocyte signatures, which were linked to treatment response and relapse rates.^[11]^ Anne *et al*. combined scRNA-seq with TCR profiling to reveal that GZMB+CD8^+^ T cells promoted macrophage infiltration and procaspase-3 activation in renal tissue, thereby worsening kidney injury.^[39]^ Shane *et al*. detected low-frequency PR3-specific B cells in PR3-seropositive patients, suggesting an early emergence of PR3 self-reactivity.^[40]^ These studies deepen our understanding of immune cell-specific expression patterns and support the development of personalized therapies.

This study has several limitations. First, the cohort size was small, comprising only three GPA patients and three HCs; as a result, comparisons between some neutrophil subtypes revealed trends without reaching statistical significance. This work was intended as a pilot investigation to characterize neutrophil heterogeneity in GPA, thereby establishing a preliminary framework for future analyses. Second, patients with MPA or EGPA were not included in our scRNA-seq analysis and subsequent comparison. This constrains comprehensive characterization of neutrophil heterogeneity across AAV, as well as the identification of subtype-specific neutrophil signatures. Third, flow cytometry validation of the identified subset was performed in few GPA patients, owing to technical challenges in staining intracellular proteins in neutrophils. Fourth, whole blood samples are dominated by neutrophils, which limited the resolution for profiling other immune cell types. Lastly, our analysis was restricted to circulating cells and did not include tissue-resident populations. Integrated analysis of both peripheral blood and lesioned tissue neutrophils is critical to fully elucidate their phenotypic dynamics and pathogenic roles in AAV. Further studies are needed to connect neutrophil phenotypes with clinical outcomes and treatment responses.

In summary, we present the first single-cell atlas of neutrophil heterogeneity in GPA, identifying distinct subsets and their molecular features. The Neu_PLA2G7 subset emerges as a promising biomarker, providing novel insights into AAV-associated hyperinflammation and potential avenues for personalized therapy and disease monitoring.

## Supplementary information

*Supplementary materials are only available at the official site of the journal (www.rir-journal.com)*.

## Supplementary Material

Supplementary Material Details
