# Supplementary material for: Single-cell RNA sequencing highlights the heterogeneity of neutrophils in granulomatosis with polyangiitis
Source: Rheumatol Immunol Res. 2026 Oct 22;7(3):211–23. doi: 10.1515/rir-2026-0032 (PMC13689950; doi:10.1515/rir-2026-0032)
Supplement: Supplementary file 1 [file rir-2026-0032_sm.pdf]

Supplementary materials

Supplementary Figures

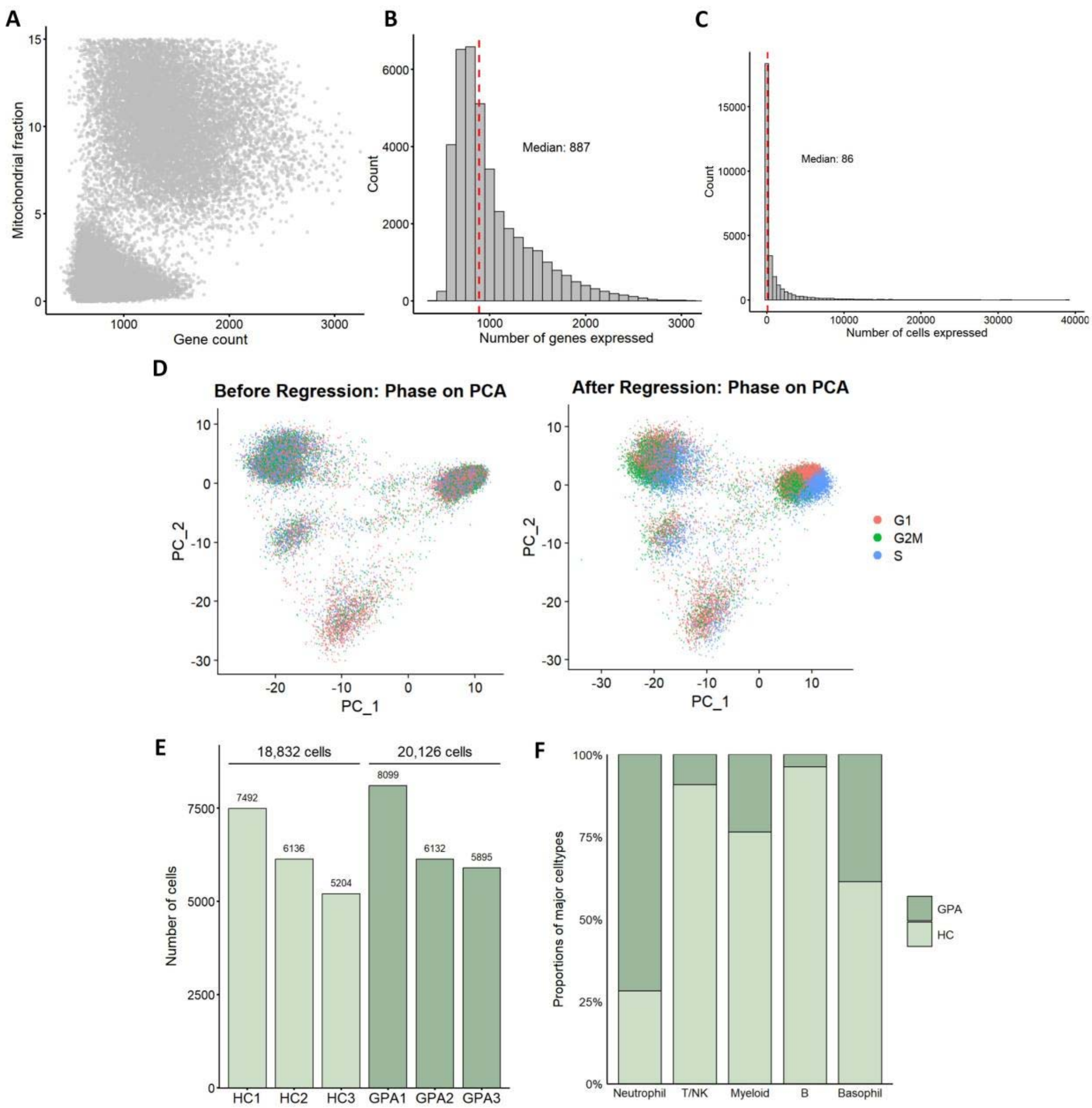

Supplementary Figure 1. Quality control and cell composition in scRNA-seq data of peripheral blood cells. (A) Scatter plot displaying the mitochondrial fractions across all cells (dots), after excluding cells with a high mitochondrial content (cutoff: 15%). (B) Distribution of the number of genes detected per cell in the scRNA-seq dataset. (C) Distribution of the number of cells expressing the identified genes in the scRNA-seq data. (D) PCA plots showing cells colored by

predicted cell cycle phase (G1, S, G2M), before (left) and after (right) regression of cell cycle scores. (E) Cell counts (Y-axis) measured across six individual samples (X-axis). (F) Proportion of major cell types in the GPA and HC groups.

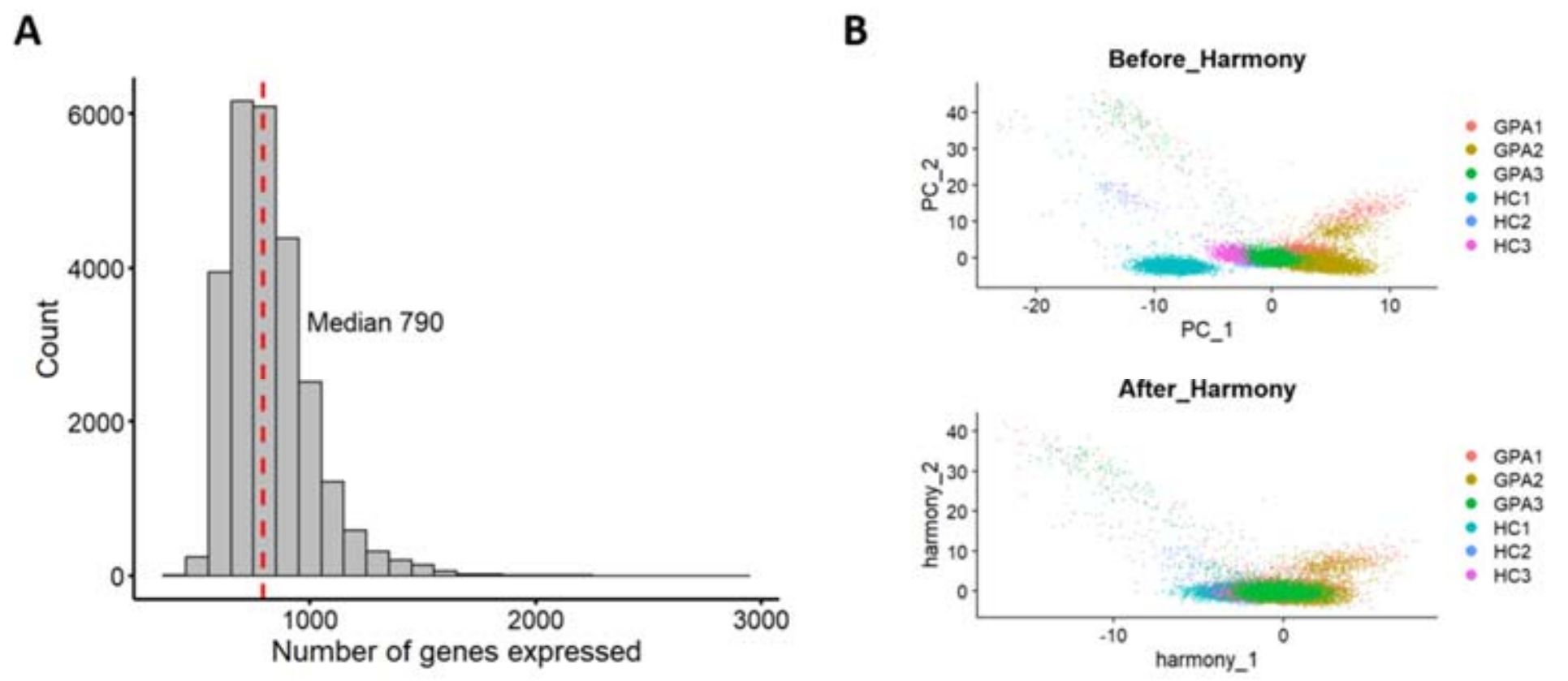

Supplementary Figure 2. Characteristics of gene expression in neutrophils and batch effect removal. (A) Distribution of the number of genes detected per cell in neutrophils. (B) Application of the harmony method to remove batch effects in neutrophil populations across different samples.

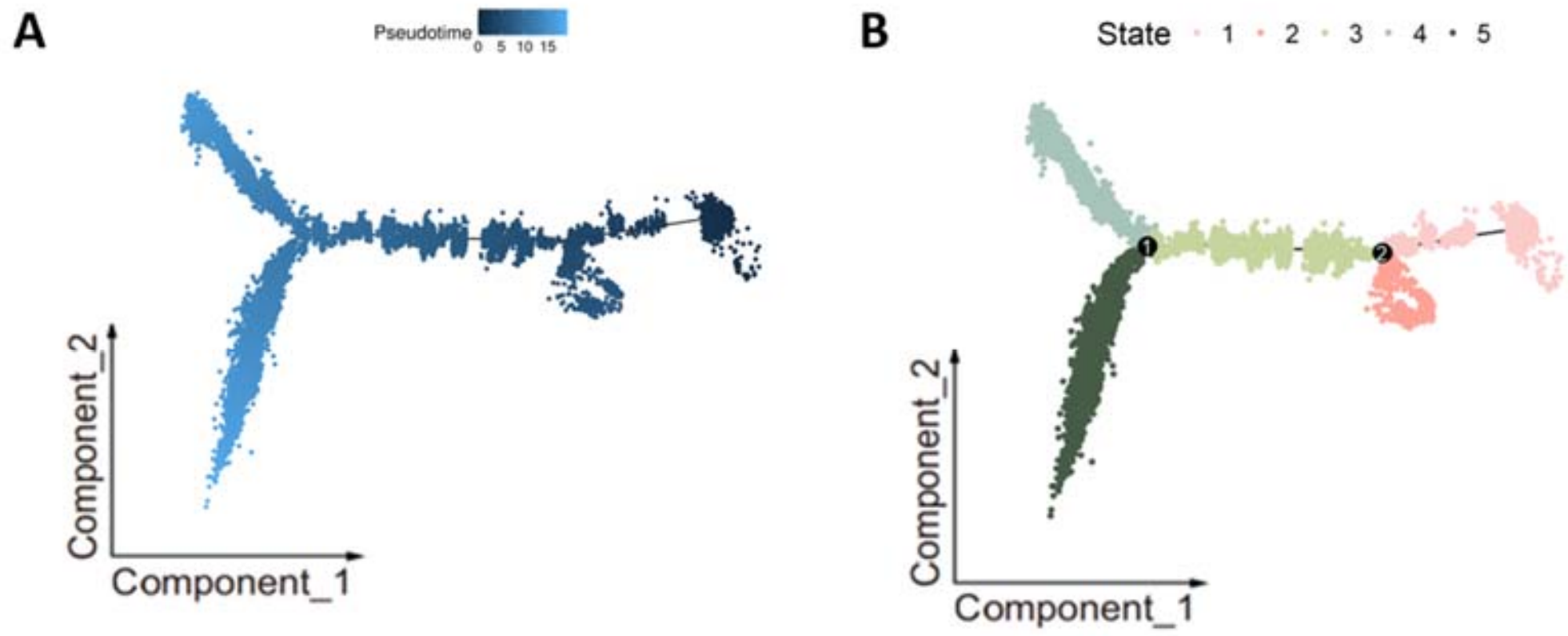

Supplementary Figure 3. Pseudo-time trajectory of neutrophils. (A and B) Pseudo-time trajectory of neutrophils, colored by pseudotime (A) and state (B).

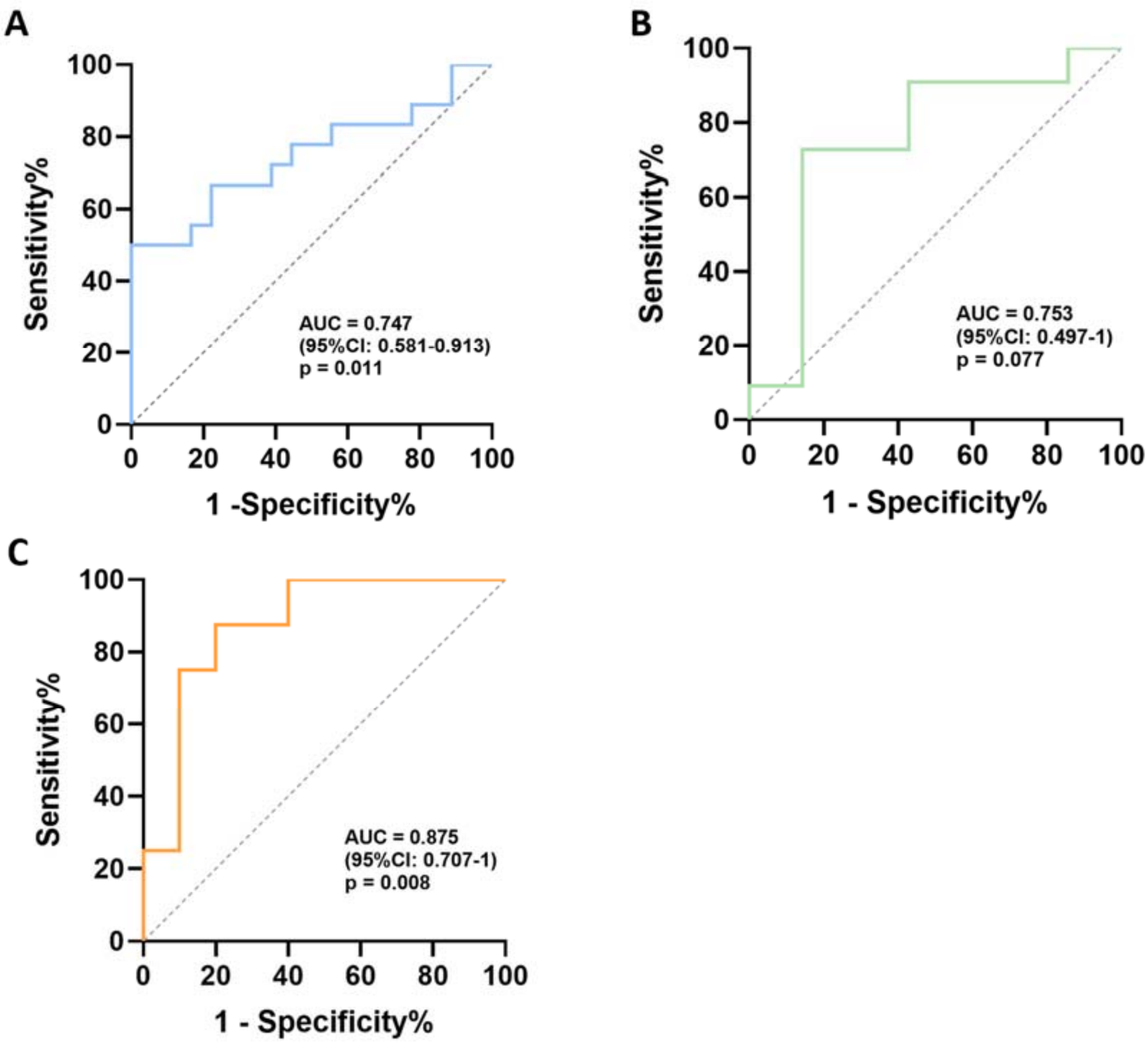

Supplementary Figure 4. Clinical efficacy of the relative RNA levels of PLA2G7 in neutrophils. (A-C) ROC curve illustrating the predictive value of relative RNA levels of *PLA2G7* in neutrophils for identifying AAV patients compared to healthy individuals (A), distinguishing those with renal involvement from those without (B), and differentiating exacerbation or relapse AAV patients from those in remission or stabilization (C).

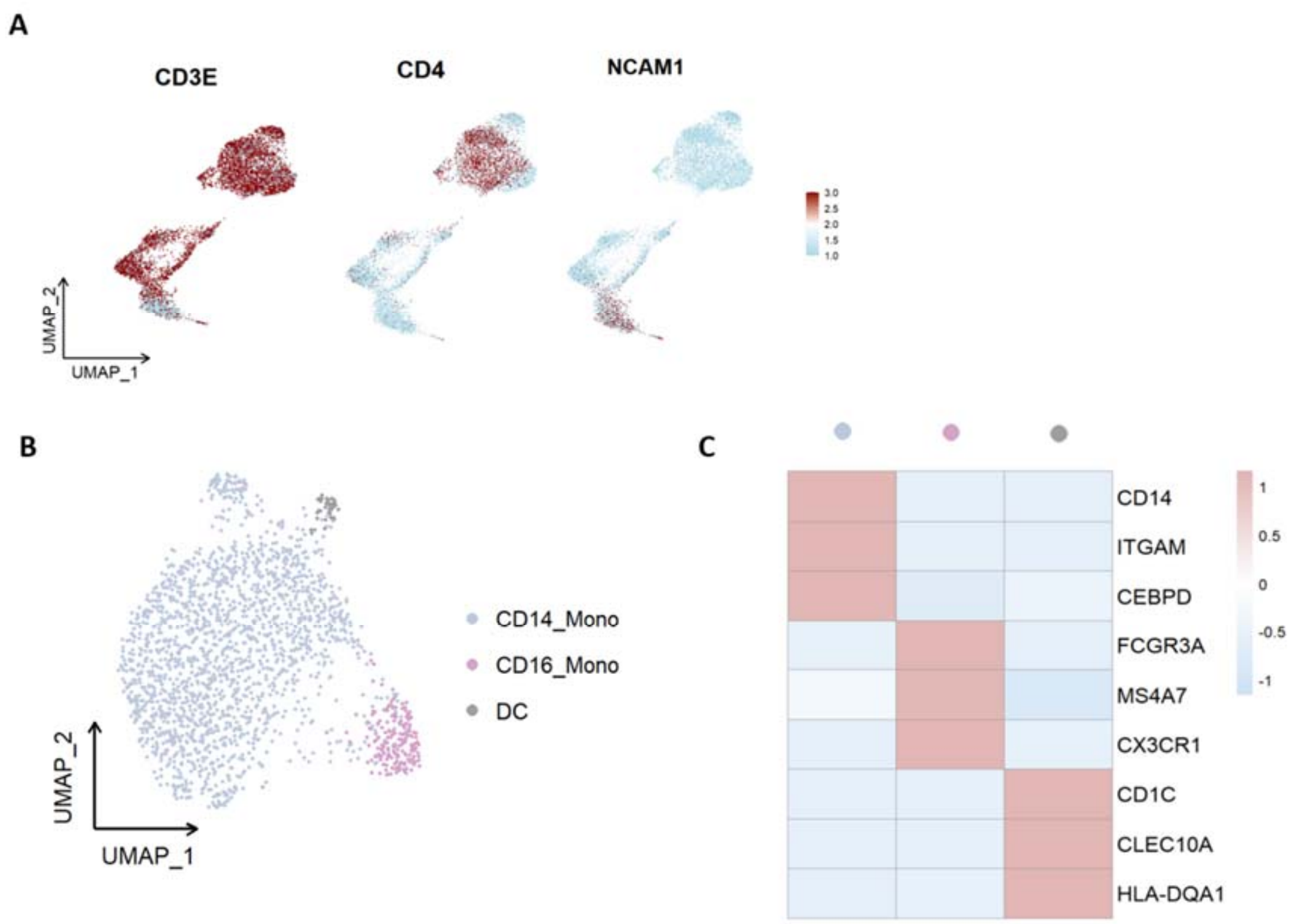

Supplementary Figure 5. Heterogeneity of myeloid subtypes in GPA. (A) Feature plots of canonical marker genes of T cell. (B) UMAP plot depicting the distribution of circulating myeloid cells in GPA patients (*n* = 3) and healthy controls (HCs, *n* = 3), with color coding based on the identified subpopulations. (C) Heatmap showing the normalized average expression of monocyte or dendritic cell function-associated genes across each cell subpopulation.

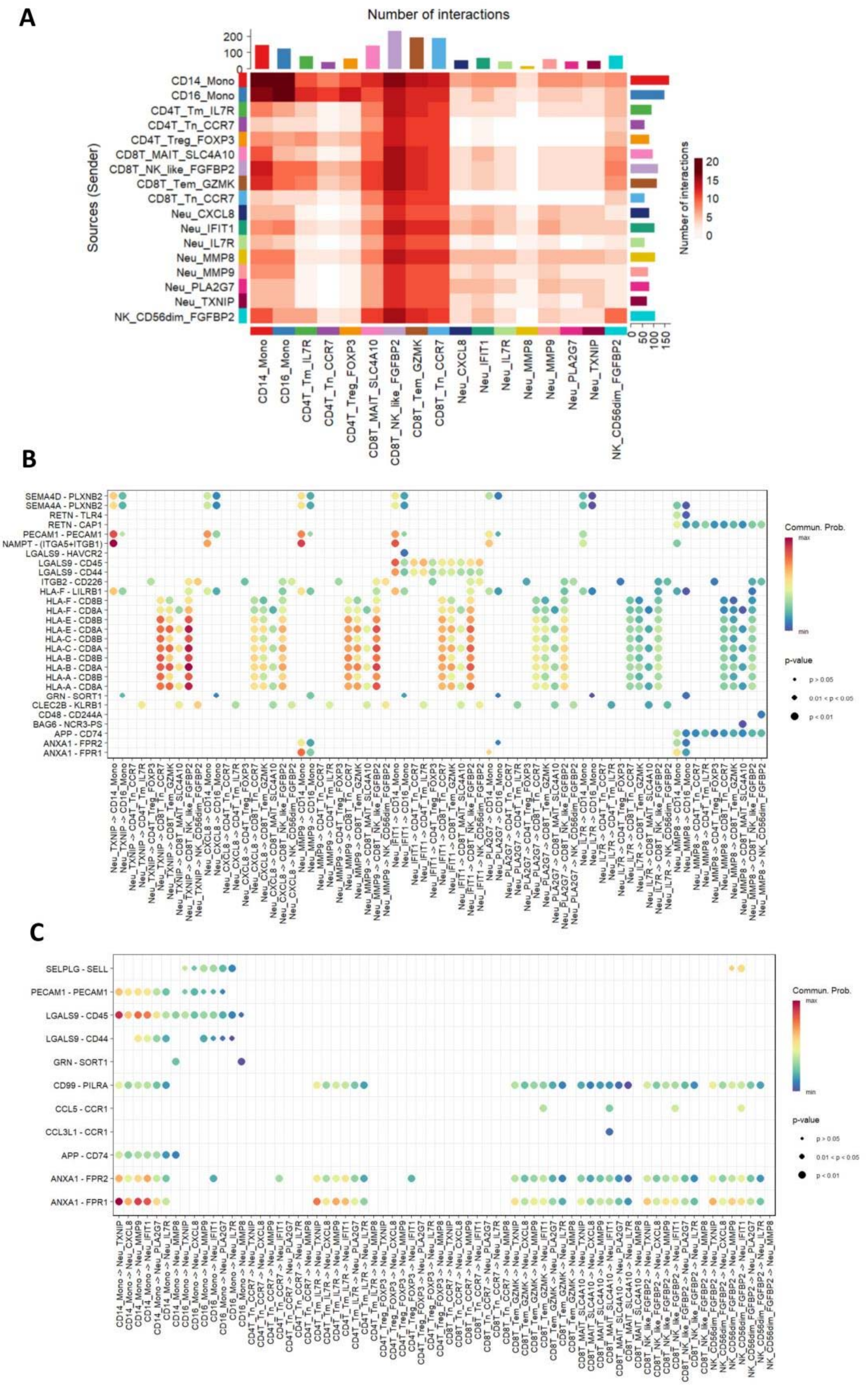

Supplementary Figure 6. Communication between neutrophils and other immune cells. (A) Heatmap illustrating the number of interactions between different immune cell types in the periphery. (B and C) Dot plot illustrating the

signaling from neutrophil subsets to other immune cell populations (B), and from other immune cell populations to neutrophil subsets (C). Each point represents an interaction between immune cells, with point size and color indicating the probability of communication.

Supplementary Table 1. Characteristics of AAV patients recruited for for scRNA-seq and RT-qPCR

Three GPA patients recruited for scRNA-seq

	Patient		GPA1	GPA2	GPA3
	Age		47	70	33
	Sex		Male	Female	Female
	New/Worse/Persistent		New	New	Worse
BVAS	General		0	0	0
	Cutaneous		0	0	0
	Mucous membranes/Eyes		4	3	0
	ENT		4	10	4
	Chest		3	3	4
	Cardiovascular		0	0	0
	Renal		0	0	0
	Nervous system		1	0	1
	SUM		12	16	9
Blood Count	White Blood Cells	× 10^9 /L	10.15	6.12	9.56
	Neutrophils	%	76.4	67.7	77.0
	Lymphocytes	%	13.1	17.3	16.2
	Monocytes	%	8.6	7.5	4.6
	Eosinophils	%	0.3	6.7	0.07
	Hemoglobin	g/L	121	131	131
Urine	Red Blood Cell	Cells/ul	Negative	Negative	Negative
	Protein	g/L	Negative	Negative	Negative
Biochemical	ALT	U/L	18	14	14
	ALB	g/L	14	41	42
	Creatinine	umol/L	66	49	50
	Urea	mmol/L	4.55	2.66	3.57
	ESR	mm/h	75	55	40
	hsCRP	mg/L	15.5	17.0	18.02
Immunological	PR3-ANCA	AU/mL	2.5	76.5	< 1.5
	MPO-ANCA	AU/mL	< 1.5	< 1.5	< 1.5

Eleven GPA patients and seven MPA patients recruited for RT-qPCR

Characteristics		GPA Patients (*n* = 11)	MPA Patients (*n* = 7)
Age at diagnosis, Median (IQR), years		37 (23, 54)	43 (27, 68)
Sex, male/female, number		8/3	6/1
Exacerbation/Relapse, *n* (%)		6 (54.5)	4 (57.1)
Clinical manifestations	General, *n* (%)	1 (9.1)	3 (42.9)
	Cutaneous, *n* (%)	3 (27.3)	4 (57.1)
	Mucous membranes/Eyes, *n* (%)	5 (45.4)	0
	ENT, *n* (%)	6 (54.5)	0
	Chest, *n* (%)	6 (54.5)	3 (42.9)
	Cardiovascular, *n* (%)	0	0
	Renal, *n* (%)	4 (36.4)	3 (42.9)
	Nervous system, *n* (%)	0	0
Biochemical	ALT, Median (IQR), U/L	23 (9, 42)	14 (6, 21.4)
	ALB, Median (IQR), g/L	34 (26, 47)	43.8 (21, 49)
	Creatinine, Median (IQR), umol/L	69 (51, 95)	75 (52, 92)
	Urea, Median (IQR), mmol/L	8 (4.4, 59)	20.5 (9.3, 45.9)
	ESR, Median (IQR), mm/h	19 (2, 73)	23.5 (7.8, 42.7)
	hsCRP, Median (IQR), mg/L	7.6 (3.9, 39.1)	5 (0.7, 84)
Immunological	PR3-ANCA, AU/mL	98,2 (26.3, 338.8)	-
	MPO-ANCA, AU/mL	-	177.5 (1.5, 1397)

Supplementary Table 2. Top 30 upregulated differentially expressed genes in neutrophil subtypes comparing GPA patients to HCs

Gene	Adjusted P value	Log2 Fold Change	Cluster
TXNIP	0	0.657	Neu_TXNIP
H3-3A	0	0.548	Neu_TXNIP
RIPOR2	0	0.503	Neu_TXNIP
CXCR2	0	0.459	Neu_TXNIP
MALAT1	0	0.324	Neu_TXNIP
TLR2	1.88285790676432e-299	0.719	Neu_TXNIP
SMAP2	7.50656963956048e-274	0.463	Neu_TXNIP
PTPRC	4.61763518876128e-209	0.359	Neu_TXNIP
ARHGAP15	1.57175492736015e-193	0.795	Neu_TXNIP
ENSG00000288700	1.4391353984522e-187	0.818	Neu_TXNIP
SCLT1	1.24379471445494e-162	0.733	Neu_TXNIP

CYRIB	4.76080618164613e-162	0.534	Neu_TXNIP
ADGRE2	2.68583011148647e-161	0.608	Neu_TXNIP
TSC22D3	5.38706271266909e-151	0.421	Neu_TXNIP
SSH2	2.5761894741439e-147	0.540	Neu_TXNIP
FKBP5	1.41601426013125e-140	0.590	Neu_TXNIP
VMP1	2.60275539696674e-138	0.389	Neu_TXNIP
NIBAN1	3.8043512310269e-136	0.363	Neu_TXNIP
SLC8A1	1.92452498977799e-132	0.777	Neu_TXNIP
H3-3B	3.02802304476539e-130	0.317	Neu_TXNIP
TLR1	4.43327017980231e-127	0.535	Neu_TXNIP
IL1R2	4.43109848464682e-115	0.409	Neu_TXNIP
SORL1	2.13683951584933e-113	0.390	Neu_TXNIP
PELATON	5.32931072583538e-112	0.504	Neu_TXNIP
ENSG00000228655	3.25614205890804e-111	0.759	Neu_TXNIP
TBXAS1	3.49870624192015e-111	0.554	Neu_TXNIP
IL1RAP	1.14183981529607e-110	0.667	Neu_TXNIP
FCGR2A	2.59843718286334e-103	0.332	Neu_TXNIP
PELI1	7.06873495034751e-100	0.406	Neu_TXNIP
PAG1	1.19508498438566e-99	0.606	Neu_TXNIP
H3F3A	0	3.446	Neu_CXCL8
H3F3B	0	3.034	Neu_CXCL8
CXCL8	0	2.288	Neu_CXCL8
NFKBIA	0	2.263	Neu_CXCL8
G0S2	0	2.252	Neu_CXCL8
GABARAP	0	2.157	Neu_CXCL8
HBA2	0	2.059	Neu_CXCL8
IER3	0	2.023	Neu_CXCL8
ATP6V0C	0	1.934	Neu_CXCL8

SMIM25	0	1.901	Neu_CXCL8
FAM49B	0	1.731	Neu_CXCL8
GNG10	0	1.714	Neu_CXCL8
PPP1R15A	0	1.712	Neu_CXCL8
NFKBIZ	0	1.651	Neu_CXCL8
AC012368.1	0	1.552	Neu_CXCL8
HBB	0	1.545	Neu_CXCL8
IDS	0	1.487	Neu_CXCL8
CXCL1	0	1.478	Neu_CXCL8
FOSB	0	1.470	Neu_CXCL8
IFI30	0	1.451	Neu_CXCL8
ZFP36	0	1.447	Neu_CXCL8
HBA1	0	1.440	Neu_CXCL8
TNFAIP3	0	1.361	Neu_CXCL8
DUSP6	0	1.306	Neu_CXCL8
H2AFZ	0	1.299	Neu_CXCL8
HIST1H2AC	0	1.276	Neu_CXCL8
RNASEK	0	1.264	Neu_CXCL8
LRMP	0	1.253	Neu_CXCL8
AC023157.3	0	1.244	Neu_CXCL8
SGK1	0	1.163	Neu_CXCL8
MMP9	0	2.306	Neu_MMP9
S100A12	0	1.838	Neu_MMP9
PADI4	0	1.561	Neu_MMP9
RFLNB	0	1.529	Neu_MMP9
HMGB2	0	1.518	Neu_MMP9
ARG1	0	1.507	Neu_MMP9
CD177	0	1.347	Neu_MMP9
FCN1	0	1.319	Neu_MMP9
CST7	0	1.186	Neu_MMP9
APMAP	0	1.139	Neu_MMP9
ALOX5AP	0	1.129	Neu_MMP9
S100P	0	1.113	Neu_MMP9
VIM	0	1.087	Neu_MMP9
PGLYRP1	0	1.059	Neu_MMP9
PLP2	0	1.051	Neu_MMP9
CDA	0	1.039	Neu_MMP9
S100A8	0	1.033	Neu_MMP9
MTARC1	0	1.022	Neu_MMP9
H2AC6	0	1.013	Neu_MMP9
H2BC21	0	0.995	Neu_MMP9
QPCT	0	0.994	Neu_MMP9
CKAP4	0	0.976	Neu_MMP9
CR1	0	0.949	Neu_MMP9

S100A6	0	0.874	Neu_MMP9
MME	0	0.852	Neu_MMP9
S100A9	0	0.596	Neu_MMP9
ACTB	0	0.485	Neu_MMP9
PGD	1.74230907171608e-301	0.925	Neu_MMP9
PYGL	8.9326656379982e-301	0.831	Neu_MMP9
TXN	7.87380634166657e-298	0.945	Neu_MMP9
RSAD2	0	3.115	Neu_IFIT1
IFIT3	0	2.927	Neu_IFIT1
IFIT1	0	2.742	Neu_IFIT1
IFIT2	0	2.740	Neu_IFIT1
MX1	0	2.592	Neu_IFIT1
XAF1	0	2.452	Neu_IFIT1
HERC5	0	2.227	Neu_IFIT1
ISG15	0	2.180	Neu_IFIT1
OAS3	0	2.129	Neu_IFIT1
IFI44L	0	2.056	Neu_IFIT1
EPSTI1	0	2.037	Neu_IFIT1
LY6E	0	2.006	Neu_IFIT1
IFI44	0	1.915	Neu_IFIT1
PARP14	0	1.829	Neu_IFIT1
OAS1	0	1.787	Neu_IFIT1
SAMD9L	0	1.751	Neu_IFIT1
OAS2	0	1.719	Neu_IFIT1
TRIM22	0	1.710	Neu_IFIT1
LIPA	0	1.708	Neu_IFIT1
GBP1	0	1.655	Neu_IFIT1
FAM3B	0	1.603	Neu_IFIT1
PLSCR1	0	1.521	Neu_IFIT1
DDX60	0	1.498	Neu_IFIT1
IFIH1	0	1.410	Neu_IFIT1
IFI16	0	1.400	Neu_IFIT1
APOL6	0	1.385	Neu_IFIT1
SAMD9	0	1.381	Neu_IFIT1
CMPK2	0	1.365	Neu_IFIT1
MT2A	0	1.357	Neu_IFIT1
OASL	0	1.356	Neu_IFIT1
SMPX	0	5.282	Neu_PLA2G7
CFAP58	0	5.012	Neu_PLA2G7
ENSG00000287469	0	4.817	Neu_PLA2G7
ENSG00000288075	0	4.622	Neu_PLA2G7
EML4-AS1	0	4.089	Neu_PLA2G7

BMP7	0	4.037	Neu_PLA2G7
CDADC1	0	3.896	Neu_PLA2G7
ERBB4	0	3.634	Neu_PLA2G7
ZNF19	0	3.627	Neu_PLA2G7
THEMIS	0	3.594	Neu_PLA2G7
ENSG00000254178	0	3.444	Neu_PLA2G7
MIR4300HG	0	3.413	Neu_PLA2G7
ENSG00000242880	0	3.396	Neu_PLA2G7
ENSG00000227042	0	3.392	Neu_PLA2G7
ENSG00000287372	0	3.389	Neu_PLA2G7
LINC01320	0	3.276	Neu_PLA2G7
RFTN1	0	3.251	Neu_PLA2G7
TMPRSS5	0	3.211	Neu_PLA2G7
WDR19	0	3.199	Neu_PLA2G7
PLA2G7	0	3.199	Neu_PLA2G7
ENSG00000214870	0	3.098	Neu_PLA2G7
LINC00486	0	3.029	Neu_PLA2G7
ENSG00000287394	0	2.993	Neu_PLA2G7
NGB	0	2.890	Neu_PLA2G7
PCAT1	0	2.862	Neu_PLA2G7
HNF4G	0	2.711	Neu_PLA2G7
ENSG00000284377	0	2.697	Neu_PLA2G7
SWAP70	0	2.691	Neu_PLA2G7
PDE1C	0	2.615	Neu_PLA2G7
TTLL5	0	2.608	Neu_PLA2G7
KLF12	0	1.963	Neu_IL7R
FYN	0	1.958	Neu_IL7R
RORA	0	1.601	Neu_IL7R
IL7R	8.89245161728668e-289	2.285	Neu_IL7R
TC2N	1.40900481112207e-261	1.134	Neu_IL7R
TCF7	2.26731900364724e-251	1.512	Neu_IL7R
CAMK4	6.24335743072337e-248	1.435	Neu_IL7R
PPP1R16B	1.62241908727818e-244	1.144	Neu_IL7R
DOCK10	7.7754304694261e-216	1.674	Neu_IL7R
BCL11B	1.42177279265034e-206	1.403	Neu_IL7R
ARL4C	4.17185626108602e-200	1.774	Neu_IL7R

NAP1L1	1.74577951292581e-196	2.233	Neu_IL7R
CD96	4.29604952172941e-188	1.426	Neu_IL7R
CD99	1.05484047749024e-187	1.312	Neu_IL7R
CD2	7.72558042700802e-185	0.865	Neu_IL7R
BCL2	7.07595042489078e-184	1.367	Neu_IL7R
TLE5	7.22472523461182e-181	1.568	Neu_IL7R
ITK	1.47720306607069e-174	1.124	Neu_IL7R
STEAP1B	2.26814307688043e-172	0.861	Neu_IL7R
DIPK1A	6.9674893071781e-170	0.957	Neu_IL7R
SKAP1	2.39251533569761e-169	1.480	Neu_IL7R
CD247	3.38028877243721e-168	1.546	Neu_IL7R
INPP4B	8.70534729009149e-166	1.348	Neu_IL7R
CD69	5.84362274758183e-159	1.229	Neu_IL7R
CD226	8.27703968808742e-158	0.972	Neu_IL7R
GPRIN3	2.17214236009362e-150	1.207	Neu_IL7R
IKZF3	4.59628548303347e-119	0.887	Neu_IL7R
RUNX3	5.34196204943399e-114	1.517	Neu_IL7R
BACH2	9.02800557598533e-114	0.956	Neu_IL7R
AKT3	5.11628281830489e-113	1.071	Neu_IL7R
LTF	0	7.785	Neu_MMP8
LCN2	0	6.539	Neu_MMP8
DEFA3	0	6.276	Neu_MMP8
CAMP	0	5.475	Neu_MMP8
BPI	0	5.044	Neu_MMP8
OLFM4	0	5.013	Neu_MMP8
CD24	0	4.784	Neu_MMP8

CEACAM8	0	4.686	Neu_MMP8
DEFA4	0	4.635	Neu_MMP8
MMP8	0	4.355	Neu_MMP8
CRISP3	0	4.011	Neu_MMP8
MS4A3	0	3.606	Neu_MMP8
STOM	0	3.514	Neu_MMP8
CYBB	0	3.446	Neu_MMP8
TCN1	0	3.413	Neu_MMP8
RNASE3	0	3.331	Neu_MMP8
RNASE2	0	3.304	Neu_MMP8
MPO	0	3.075	Neu_MMP8
ABCA13	0	3.009	Neu_MMP8
UGCG	0	2.927	Neu_MMP8
CPNE3	0	2.878	Neu_MMP8
NKG7	0	2.868	Neu_MMP8
GPI	0	2.840	Neu_MMP8
RETN	0	2.802	Neu_MMP8
ATP8B4	0	2.785	Neu_MMP8
CEACAM6	0	2.734	Neu_MMP8
PKM	0	2.707	Neu_MMP8
ELANE	0	2.691	Neu_MMP8
RGCC	0	2.643	Neu_MMP8
PLAC8	0	2.537	Neu_MMP8

GPA: granulomatosis with polyangiitis; HCs: healthy controls.
